# Genomic and phenotypic characteristics of Swedish *C*. *jejuni* water isolates

**DOI:** 10.1371/journal.pone.0189222

**Published:** 2017-12-07

**Authors:** Anna Nilsson, Cecilia Johansson, Astrid Skarp, René Kaden, Lars Engstrand, Hilpi Rautelin

**Affiliations:** 1 Department of Medical Sciences, Clinical Microbiology, Uppsala University, Uppsala, Sweden; 2 Department of Microbiology, Tumor and Cell Biology, Karolinska Institute, and Science for Life Laboratory, Stockholm, Sweden; Massey University, NEW ZEALAND

## Abstract

*Campylobacter jejuni* is the most common cause of bacterial gastroenteritis. Major reservoirs are warm-blooded animals, poultry in particular, but *Campylobacter* can also be transmitted via water. In this paper, we have taken a closer look at the biology and potential virulence of *C*. *jejuni* water isolates. Seven *C*. *jejuni* isolates from incoming surface water at water plants in Sweden were characterized with whole genome sequencing and phenotypical testing. Multi locus sequence typing analysis revealed that these isolates belonged to groups known to include both common (ST48CC) and uncommon (ST1275CC, ST683, ST793 and ST8853) human pathogens. Further genomic characterization revealed that these isolates had potential for arsenic resistance (due to presence of *arsB* gene in all isolates), an anaerobic dimethyl sulfoxide oxidoreductase (in three isolates) and lacked the MarR-type transcriptional regulator gene *rrpB* (in all but one isolate) earlier shown to be involved in better survival under oxidative and aerobic stress. As putative virulence factors were concerned, there were differences between the water isolates in the presence of genes coding for cytolethal distending toxin (*cdt*ABC), Type VI secretion system and sialylated LOS, as well as in biofilm formation. However, all isolates were motile and could adhere to and invade the human HT-29 colon cancer cell line *in vitro* and induce IL-8 secretion suggesting potential to infect humans. This is, to the best of our knowledge, the first study where *C*. *jejuni* water isolates have been characterized using whole genome sequencing and phenotypical assays. We found differences and shared traits among the isolates but also potential to infect humans.

## Introduction

*Campylobacter* is the most common cause of bacterial gastroenteritis in most parts of the world and *C*. *jejuni* is the predominant pathogen [1]. Warm-blooded animals, such as poultry, pigs and ruminants are major reservoirs for *Campylobacter* and the bacteria are thought to be mainly transmitted via handling and eating raw or undercooked meat [2,3]. *Campylobacter* can also be transmitted via environmental pathways, such as water, and waterborne outbreaks of *Campylobacter* are not uncommon [4–7]. In the Nordic countries, groundwater for drinking water is usually not treated and reports from Finland describe *Campylobacter* outbreaks where heavy rain has led to contamination of groundwater wells [6,7]. It has also been shown that cattle drinking untreated water from lakes or private water supplies are more likely to test positive for *Campylobacter* [8,9]. Studies have shown *C*. *jejuni* water survival time at a low temperature to vary between two weeks and four months [10–13] and strains isolated from different sources have been noted to show different survival potentials [13–15]. These inter-strain differences have been suggested to be caused by variations in genetic content [12], however, whole genome sequencing has rarely been used to study waterborne *C*. *jejuni*.

The population structure of *C*. *jejuni* is highly diverse and described using Multi Locus Sequence Typing (MLST). To date there are almost 9000 STs, divided into several clonal complexes (CCs; http://pubmlst.org/campylobacter/, last accessed 22 May 2017). The CCs of *C*. *jejuni* can be considered to consist of generalists when able to colonize various hosts or specialists if host- or niche-specific [16].

*C*. *jejuni* are fastidious bacteria needing a microaerobic environment for survival and growth, which optimally occurs at 42°C. However, *C*. *jejuni* have been isolated from environmental sources where neither the atmosphere nor the temperature have been optimal [11–13]. For survival in the environment, bacteria can use strategies such as biofilm formation and motility [17,18]. By forming biofilm, bacteria can provide themselves with a protective environment under harsh conditions and also allow for dispersal of the bacteria. Motility has been shown to be important for biofilm formation and the ability to form biofilm has been noted to vary between different lineages of *C*. *jejuni* [19–21].

To study the potential of *Campylobacter* to cause human infections, *in vitro* infection models with human epithelial cell lines are frequently used to determine adherence, invasion and downstream cellular responses. Several studies have demonstrated differences between *C*. *jejuni* strains in the ability to adhere to and invade cell lines *in vitro* and it has been suggested that *C*. *jejuni* strains causing more severe symptoms also adhere to and invade cells *in vitro* at a higher grade than strains causing milder symptoms [22]. However, other studies have shown that both *C*. *jejuni* strains causing mild and those causing severe human infections adhere to and invade cell lines *in vitro* to a similar extent [23]. *C*. *jejuni* have also been shown to be capable of inducing downstream cell responses such as interleukin 8 (IL-8) secretion [24,25], an early signal for acute inflammatory response to bacterial infection [24]. The IL-8 response caused by *C*. *jejuni* has been suggested to be correlated to the capability of the strains to adhere and invade [24].

In this study, we characterized *C*. *jejuni* water isolates using a genotypic and phenotypic approach in order to determine features important for water survival and to reveal the potential of the isolates to infect human cells. For these purposes, whole genome sequencing, phenotypic analyses for motility and biofilm formation and an *in vitro* infection model were used.

## Material and methods

### Bacterial isolates

Seven *Campylobacter* isolates collected by the National Food Agency in 2000 from raw (incoming) surface water at water plants in Sweden were characterized in this study (Table 1). The isolates were identified as *C*. *jejuni* using MALDI-TOF Biotyping (Microflex, Bruker, Billerica, Massachusetts, US). In addition, the earlier characterized clinical strains *C*. *coli* 76339 [26] and *C*. *jejuni* 76577 [19] and the *C*. *jejuni* reference strains NCTC 11168 and 81–176 were used as experimental controls.

**Table 1:** Water sample collection information and genetic description for the *C*. *jejuni* water isolates.

*C*. *jejuni* isolate	Information on water sample collection			Genetic description				
	Location	Date	Water temp. (*°*C)	Sequence Type (ST)	Clonal Complex (CC)	Genome size (Mbp)	No. of contigs	Accession number (NCBI database)
VA1	Lerum	March, 2000	2.6	48	48	1.7*	48	NAAE00000000
VA12	Lidköping	April, 2000	4	637	1275	1.91	79	NAAF00000000
VA25	Karlshamn	September, 2000	16.6	48	48	1.66*	35	NAAG00000000
VA33	Norrköping	October, 2000	13.3	683	ua^#^	1.61	37	NACJ00000000
VA48	Karlshamn	November, 2000	7.2	793	ua^#^	1.75	63	NACK00000000
VA49	Växjö	November, 2000	6.6	8853	ua^#^	1.86	91	NACL00000000
VA52	Botkyrka	September, 2000	14.8	48	48	1.72*	21	NAAH00000000

*Plasmids included

^#^unassigned to any clonal complex

### Genomics

The isolates were cultured for 24-48h on blood agar (Columbia agar plates supplemented with 5% horse blood; Oxoid, Basingstoke, UK) in a microaerobic atmosphere (Campygen, Oxoid) at 42°C. The DNA was extracted from bacterial cultures using MagNa Pure Compact Nucleic Acid isolation kit I (Roche, Penzberg, Germany) according to manufacturer´s protocol version 12. The isolates were whole genome sequenced with an Illumina HiSeq 2500 platform with a 2 x 300 paired end run. For assembly of the reads into contigs, Geneious (version 8.1.5.) [27] with the Mira plugin (version 1.0.1.) was used and merged contigs were assembled with Geneious. The assembled sequences were annotated by RAST [28] and the translated coding sequences (tCDS) were extracted. To determine orthologues clusters, a reciprocal blastp query was performed using an E-value of 10^−5^ and the OrthAgogue [29] and MCL-edge tools [30].

Plasmids were assembled from the raw sequence reads with Geneious *de novo* assembler version 8.1.9 [27].

#### PCR

The lipooligosaccharide (LOS) locus class was determined with PCR as previously described [25] using primers described by Parker et al [31]. For the isolates that remained unassigned to a LOS locus class after PCR typing a manual search for previously sequenced LOS locus classes [32] was perfomed.

Real-time qPCR for *cdtA*, *B*, *C* and 16S rRNA was performed in the BioRad CFX96 Touch cycler using the DyNAmo HS SYBR Green qPCR kit (Thermo Scientific). The 16S rRNA primers have been previously described [33]. Primers for *cdt* genes were GGCGATGCTAGAGTTTGGC and GAACCGCTGTATTGCTCATAGG (*cdtA*), CGCGTTGATGTAGGAGCTAA and GCTCCTACATCTGTTCCTCCA (*cdtB*), and CAACAACTTCAGCTGTGCAAA and GGGGTAGCAGCTGTTAAAGGT (*cdtC*).

#### Phenotyping

All phenotypic experiments described below were performed in duplicate and repeated at least three times for each strain.

#### Biofilm

The isolates were tested for biofilm formation as previously described by Revez et al. [19] with minor modifications. A bacterial suspension with a concentration of 4 x 10^6^ CFU/ml was prepared by harvesting bacteria from blood agar and resuspending in PBS. Of the bacterial suspension, 10 μl was added to Brucella broth (Becton, Dickinson and Company, Franklin Lakes, New Jersey, US) in glass tubes and incubated at 37°C in a microaerobic environment for 48 h. After carefully removing the broth, the tubes were stained with 1% crystal violet solution. The isolates were identified as positive if a stained band was seen at the air-liquid interface. The *C*. *jejuni* strain 76577, previously identified as positive for biofilm formation [19], was used as a positive control and broth without addition of bacteria was used as a negative control.

#### Motility

The motility of the isolates was tested according to Szymanski et al. [34], with minor modifications. The isolates were cultured in Brucella broth at 42°C in a microaerobic atmosphere for 17–18 h. The bacteria were centrifuged at 8000 x g for 5 min, the supernatant removed and the pellets resuspended in PBS to a concentration of approximately 10^8^ CFU/ml. Of the bacterial suspension, 5 μl was stabbed into a Brucella soft agar plate (0.4%) and swarming zones were measured after incubation at 42°C in a microaerobic atmosphere for 48 h. The clinical *C*. *coli* clade 3 strain 76339 was included for comparison.

#### Adhesion/invasion assay

The HT-29 human colon cancer cell line (ECACC 91072201) was maintained in RPMI 1640 media (Gibco by life technologies, Carlsbad, California, US) supplemented with 2 mM glutamine (Swedish National Veterinary Institute, Uppsala, Sweden), 10% Fetal bovine serum (FBS, Gibco by Life Technologies), 100 U/ml penicillin and 100 μg/ml streptomycin (Swedish National Veterinary Institute). Overnight bacterial cultures were centrifuged, diluted in cell culture media and added to low passage HT-29 cells grown in RPMI 1640 supplemented with 1% FBS at a MOI of 100. At indicated time points, media was collected for IL-8 ELISA. For adhesion/invasion assays, cells were washed four times in PBS to remove non-adhered bacteria and lysed in 20 mM Tris, pH 7.5, 150 mM NaCl and 0.15% Triton X-100. The lysate was diluted 10 and 100 times for qPCR analysis of the 16S rRNA gene together with 10 000 times-diluted starting cultures to determine the adhesion/invasion percentage.

#### IL-8 ELISA

The IL-8 levels in the media were measured using the IL-8 ELISA Kit (Thermo Fisher Scientific, Waltham, Massachusetts, US) according to the manufacturer’s instructions. Media was diluted four to ten times prior to the assay. A standard of known concentration (included in the kit) was used to assess variations between infections. Results are expressed as fold increase over uninfected (mock) cells.

## Results

### Genomics

Seven *C*. *jejuni* isolates, collected from incoming water at water surface plants in Sweden, were whole genome sequenced (Table 1). Analysis of MLST identified ST for six of the seven isolates; ST48 (three isolates), ST637 (one isolate), ST683 (one isolate) and ST793 (one isolate), respectively. One isolate was submitted to PubMLST and assigned to the new ST8853. The ST48 isolates belonged to the ST48CC, the ST637 to ST1275CC, and the remaining three isolates were unassigned to any CC at the time of the analysis (Table 1).

To identify traits involved in virulence and survival of *C*. *jejuni* water isolates, the genomes were clustered into orthologue groups and annotated in RAST. Annotations of groups not shared by all *C*. *jejuni* water isolates, but shared by isolates from more than one ST, were manually analyzed (S1 Table). The genomes were also manually searched for genes known to be involved in specific traits of interest, such as virulence and motility.

A previously described [35] arsenic resistance gene cluster consisting of four genes (*arsP*, *arsR*, *arsC* and *acr3*) was found in the isolate VA33 (ST683) and in the three ST48 isolates. However, the gene coding for the membrane transporter *arsP* [36] was fragmented in all the four isolates and the gene coding for the efflux pump protein Acr3 was disrupted by a premature stop codon in the ST48 isolates (Table 2). The *arsP* gene was also identified in VA12 (ST1275CC, Table 2). The *arsB* gene, which is also coding for an efflux pump for arsenic [37], but not included in the gene cluster mentioned above, was detected among all of the water isolates studied (Table 2).

**Table 2:** LOS locus classes and putative virulence factors detected among the C. jejuni water isolates.

*C*. *jejuni i*solate	LOS locus class	Arsenic efflux transporters			DMSO reductase	MarR-type transcriptional regulators		Type VI secretion system	*cdtABC*
		*arsB*	*arsP*	*acr3*	*dmsABC*	*rrpA*	*rrpB*		
VA1	B2*	+	-**	-***	-	+	-	-	+
VA12	C*	+	+	-	+	+	+	+	-
VA25	B2*	+	-**	-***	-	+	-	-	+
VA33	F, J, S	+	-**	+	+	+	-	-	+
VA48	-	+	-	-	+	+	-	+	-
VA49	-	+	-	-	-	+	-	+	-
VA52	B2*	+	-**	-***	-	+	-	-	+

* potentially sialylated

**fragmented sequence

***premature stop codon

An intact gene cluster, previously described in the *C*. *jejuni* strain 81–176 [38] as coding for an anaerobic DMSO-reductase (*dmsABC*), was detected in isolates VA12 (ST1275CC), VA33 (ST683) and VA48 (ST793) (Table 2). The *dmsA* was also identified in VA49 (ST8853), but both *dmsB* and *dmsC* were disrupted. The MarR-type transcriptional regulator gene *rrpA* (regulator of the response to peroxide, Cj1546 in NCTC 11168) was found in all of the *C*. *jejuni* water isolates, however, *rrpB* (Cj1556 in NCTC 11168) was only identified in one isolate (ST1275CC; Table 2).

In three isolates, VA12 (ST1275), VA48 (ST793) and VA49 (ST8853), an intact Type VI secretion system (T6SS) gene cluster was identified (Table 2).

Intact *cdtABC* genes coding for cytolethal distending toxin (CDT) were found in the *C*. *jejuni* ST48 water isolates and in the isolate VA33 (ST683, Table 2), which was also verified with PCR (data not shown). The putative virulence genes *ciaB*, *pldA*, *cadF* and *ceuE* were identified in the genomes of all isolates, with only minor sequence variations in the predicted amino acid sequences (data not shown).

Most of the major flagellar motility genes, such as *fla*, *flg*, *fli*, *motA* and *motB*, were present in all *C*. *jejuni* water isolates. For VA12 (ST1275CC), VA33 (ST683), VA48 (ST793) and VA49 (ST8853), deletions and insertions were detected in the nucleotide sequences of the *flaA*, *flaB*, *flaG* and *flgL* genes when compared to the corresponding gene sequences in the *C*. *jejuni* reference strain NCTC 11168. However, these particular deletions and insertions did not result in any frameshifts and thus, all ORFs seemed to be intact. The *flgD*, *fliD* and *fliK* genes were detected in all *C*. *jejuni* water isolates, but variations in the sequences resulting in disrupted ORFs were detected in some of the isolates (Table 3). Furthermore, the *pseE* gene involved in flagellar modification was identified in all of the water isolates, but an intact ORF was only identified in five of the isolates (Table 3).

**Table 3:** Presence (+) or absence (-) of gene/ORF involved in flagellar motility in the *C*. *jejuni* water isolates.

Isolate	*flgD* gene/ORF	*fliD* gene/ORF	*fliK* gene/ORF	*pseE* gene/ORF
VA1	+/+	+/+	+/n.d.^a^	+/+
VA12	+/+	+/-	+/+	+/+
VA25	+/+	+/+	+/+	+/+
VA33	+/-	+/+	+/+	+/+
VA48	+/-	+/+	+/+	+/-
VA49	+/-	+/+	+/+	+/-
VA52	+/+	+/+	+/+	+/+

^a^Nucleotide sequence split over two contigs and an intact ORF could not be determined.

Two genes implicated in biofilm formation, *csrA* and *peb4*, were present in all water isolates and showed almost no differences in the gene and translated amino acid sequences. In four of the isolates, VA1 (ST48), VA25 (ST48), VA52 (ST48) and VA33), the *luxS* gene involved in quorum sensing and shown to be important for biofilm formation [39] was detected.

A plasmid of 20801 bp was identified in three of the *C*. *jejuni* water isolates (ST48CC) and annotated against the plasmid sequence of *C*. *jejuni* F38011 (S2 Table, NCBI Acc. No. CP006851), which gave the best hit (99.4%) when the sequence was blasted against all available entries in the NCBI database. Among the annotations an oxidoreductase (7-alpha-hydroxysteroid dehydrogenase) and a betalactamase were identified as well as genes possibly involved in virulence, such as prevent-host-death protein and ABC transporter permease.

LOS locus classes were assigned to five of the seven *C*. *jejuni* water isolates using PCR (data not shown). The three ST48 isolates were assigned to LOS locus class B2, VA12 (ST1275CC) to LOS locus class C and VA33 (ST683) to LOS locus class F, J or S whereas the two isolates VA48 (ST793) and VA49 (ST8853) were untypeable (Table 2). The isolates VA48 and VA49 remained unassigned even after a manual search for previously sequenced LOS locus classes.

### Phenotyping

**Fig 1:**
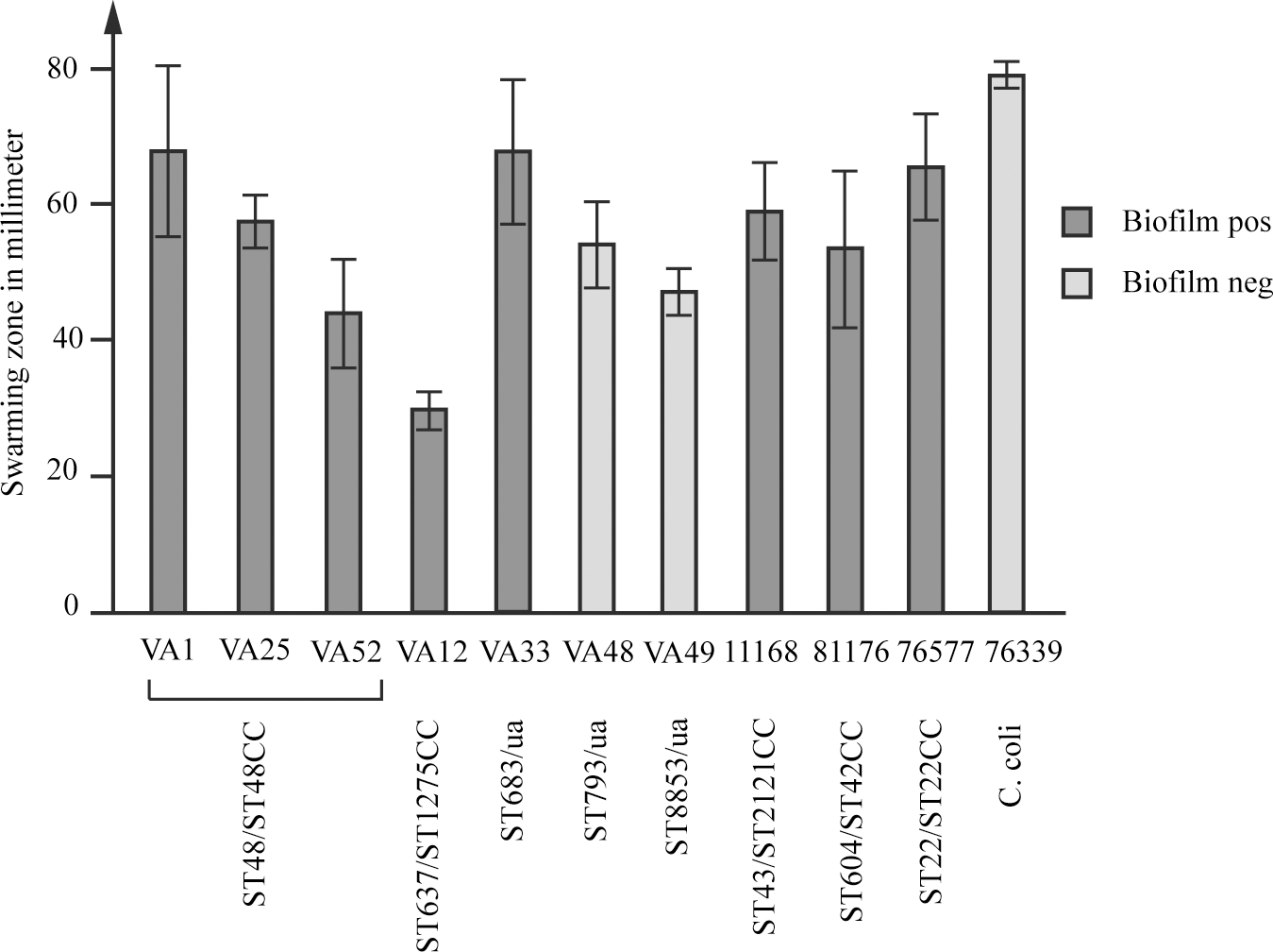
Motility and biofilm formation of the *C*. *jejuni* water isolates. Motility shown as swarming diameters in soft agar plates. Mean values of 3 experiments with error bars indicating SDs are shown. Biofilm positive isolates shown in dark grey and negative isolates in light grey. The *C*. *jejuni* strain 76577 was included as a positive control for biofilm formation. The *C*. *jejuni* strains NCTC 11168 and 81–176 and the *C*. *coli* strain 76339 were included for comparison. ST types and CCs are shown where available (ua = unassigned).

The ability of the water *C*. *jejuni* isolates to form biofilm was tested in three independent experiments and the isolates belonging to ST48 (ST48CC), ST637 (ST1275CC) and ST683 (CC ua) were able to form biofilm (Fig 1).

**Fig 2:**
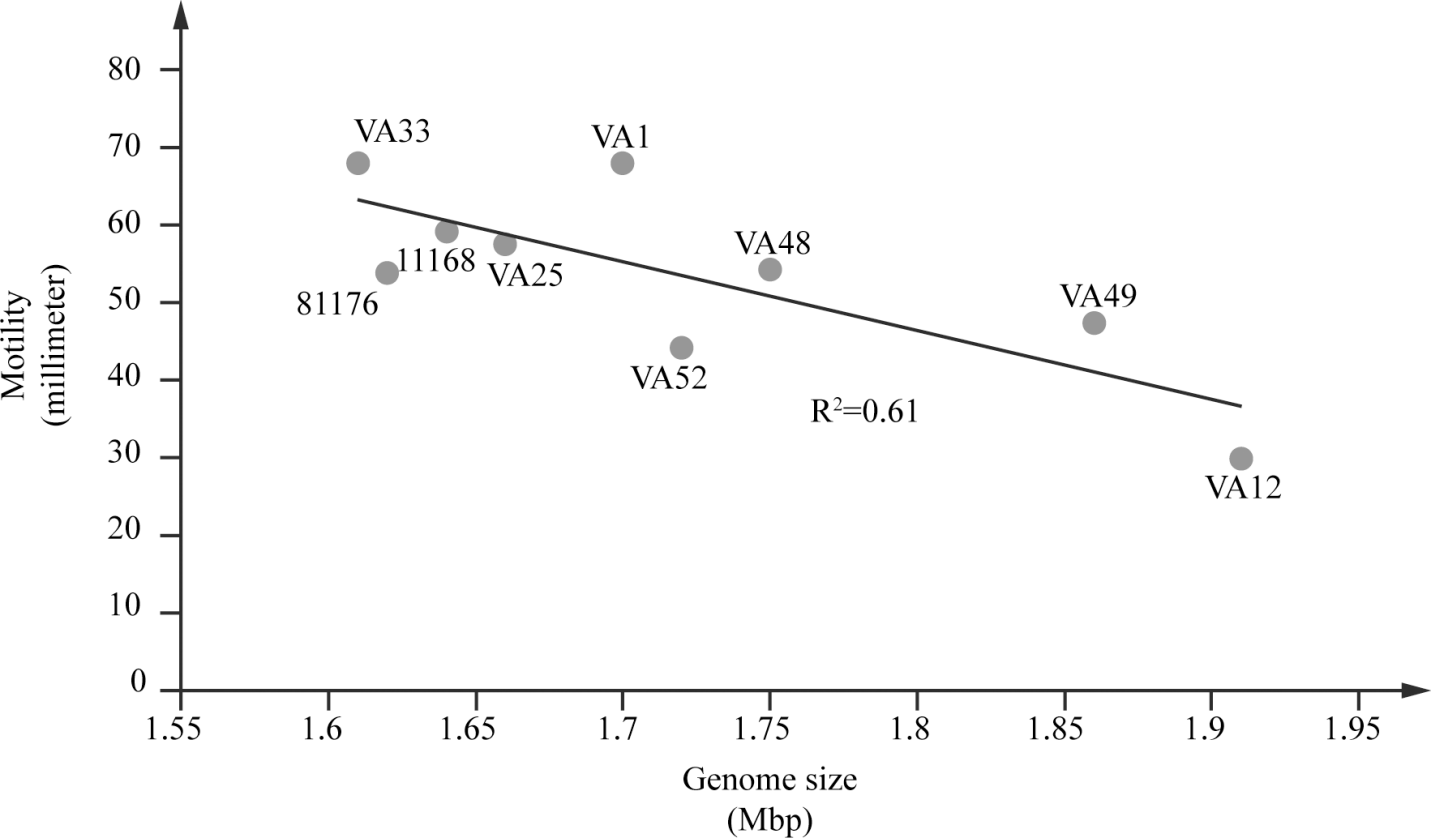
Motility and genome size of *C*. *jejuni* water isolates. Comparison of motility and genome size of the seven *C*. *jejuni* water isolates. Plasmids are included in the genome size. *C*. *jejuni* reference strains 81–176 and NCTC 11168 were included for comparison.

The motility assay showed that all isolates were motile, however, VA12 (ST1275CC) displayed the lowest motility (Fig 1). When the motility results were compared with the genome sizes, it was shown that isolates with larger genomes were less motile and a higher motility was detected among the isolates with smaller genomes (R^2^ = 0.61; Fig 2).

**Fig 3:**
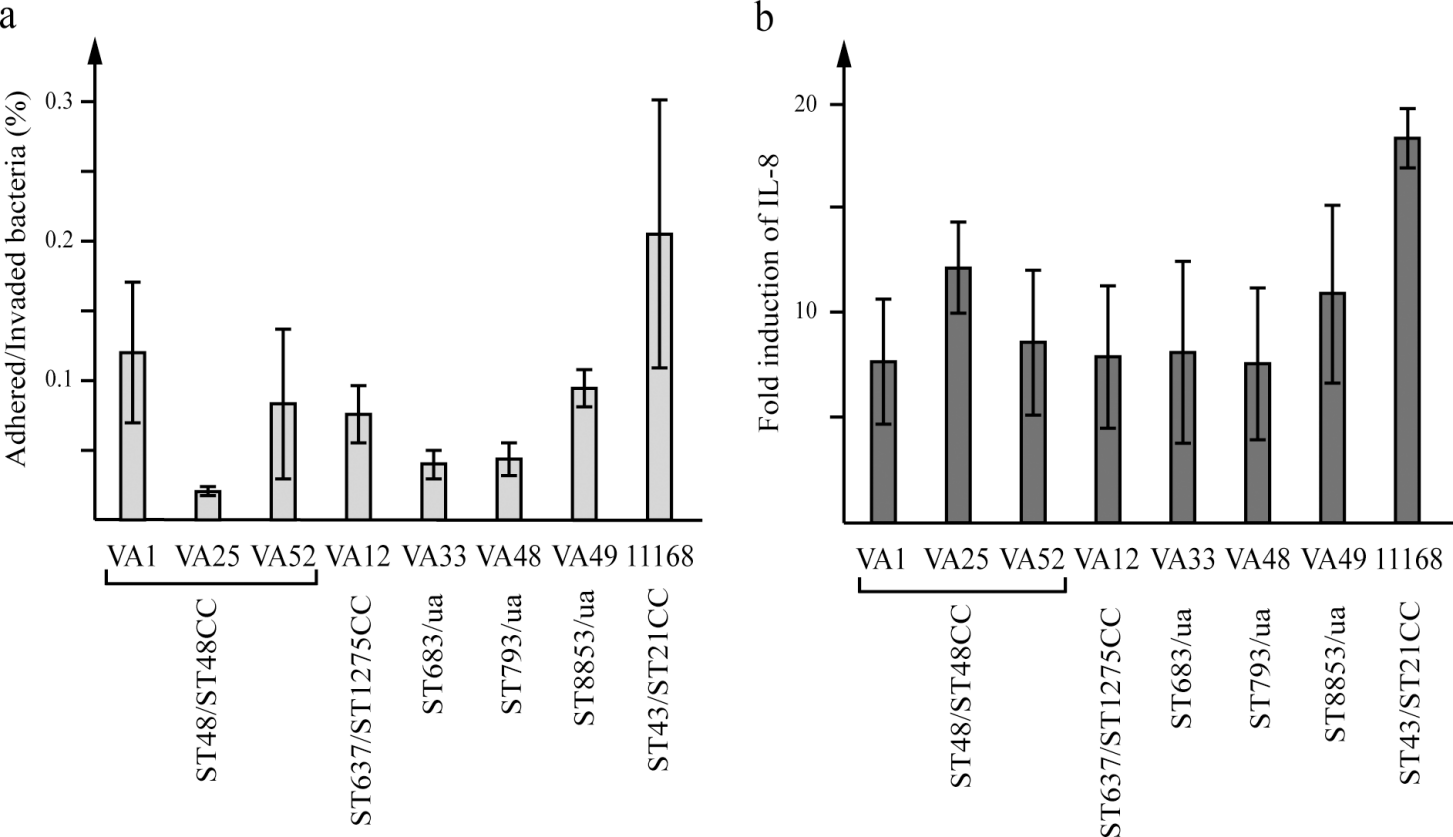
Adherence/Invasion and IL-8 induction of *C*. *jejuni* water isolates. (a) The adherence/invasion of *C*. *jejuni* water isolates to HT-29 cells 1 h post infection shown as percentage of the starting culture. (b) The induction of IL-8 levels at 2 h post infection shown as fold increase over uninfected cells. Mean values of three independent infections with error bars indicating SDs are shown. ST type and CCs shown where available (ua = unassigned).

To assess the potential virulence of the water *C*. *jejuni* isolates and see whether they would be able to infect human cells, an *in vitro* infection model with HT-29 colon cancer cells was used. All isolates were able to infect HT-29 cells (Fig 3A), and adhered bacteria could be detected already at 30 minutes post-infection (data not shown). At 1 h, mean levels ranged between 0.02 and 0.12% for the *C*. *jejuni* water isolates (Fig 3A). The level of adherence/invasion for the isolates belonging to ST48 varied among the isolates assigned to this ST. At 8 h, the levels had reached 1% for some strains (data not shown), possibly due to bacterial growth. The levels of adhered/invaded bacteria of the water isolates were consistently lower than those of the *C*. *jejuni* NCTC 11168 reference strain at all time points.

To further assess the potential ability of the water isolates to cause inflammation, IL-8 levels in the cell media were measured. IL-8 started to be detected at 2 hours post-infection and increased during the course of the infection (data not shown). In general, all water isolates induced lower levels of IL-8 than NCTC 11168 and no significant differences could be seen between the water isolates (Fig 3B). In conclusion, no clear correlation could be seen between the levels of adhered/invaded bacteria and induced IL-8 levels.

## Discussion

In this study, whole genome sequencing, phenotypical tests and *in vitro* infection results were used to characterize *C*. *jejuni* isolates collected from raw surface water at water plants in Sweden. The aim was to detect unique traits among these isolates to gain a better understanding of environmental survival of *C*. *jejuni* and the potential of the water isolates to cause human infection. Our results showed that the seven *C*. *jejuni* water isolates were genetically diverse and belonged to ST48CC (three isolates), ST1275CC (one isolate) or were unassigned to any CC (three isolates). ST48CC is one of the most common CCs among human *C*. *jejuni* isolates but also contains isolates from various other sources, including the environment [40,41]. In contrast, ST1275CC isolates have been reported to be commonly found in environmental waters and wild birds [40,42,43]. Furthermore, a search in the *Campylobacter* PubMLST database showed that ST1275CC isolates had been detected from wild birds and environmental waters, but only rarely from human samples. Moreover, ST683 and ST793, unassigned to any CC, had previously been found in samples from environmental waters and wild birds only, according to PubMLST database. Thus, according to the MLST, our water isolates included both common and uncommon human pathogens.

Whole genome sequencing revealed the gene *arsB*, coding for an efflux transporter for inorganic arsenic, in all our *C*. *jejuni* water isolates. In four of our *C*. *jejuni* water isolates (ST683 and ST48) an arsenic resistance gene cluster associated with high level resistance to inorganic arsenic [35] and with a fragmented *arsP* gene was identified. In addition, the *arsP* gene coding for an efflux transporter specific for organic arsenic [36] was found in VA12 (ST1275CC). Toxic arsenic exists in the natural environment and in order to survive in its presence bacteria have developed resistance mechanisms [35]. Organoarsenic compounds have also previously been used as growth promoters in poultry production and as antimicrobials to control coccidiosis caused by parasites [44]. However, in this study, we did not test the resistance of our isolates to arsenic compounds and therefore the role of the genes described remains to be studied.

For *Campylobacter* to survive in water, it would be beneficial to handle oxidative and aerobic stress. The MarR-type transcriptional regulator gene *rrpB* has previously been shown to be involved in *C*. *jejuni* oxidative and aerobic stress response [45]. Wildtype strains lacking *rrpB* have been shown to survive better under oxidative and aerobic stress than strains with *rrpB* [46]. The *rrpB* has also been shown to be more uncommon in *C*. *jejuni* strains associated with water and wildlife than other strains associated with bovines [46]. In our study, *rrpB* was only present in isolate VA12 (ST1275, Table 2), which suggests that this particular isolate might be more sensitive to oxidative and aerobic stress but otherwise water isolates would be more adapted to handle oxidative and aerobic stress. However, further testing is needed to confirm this finding.

DMSO exists naturally in freshwater and marine environments, in rainwater and in the atmosphere [47]. *C*. *jejuni* have been shown to be capable of utilizing DMSO as an electron acceptor under low-oxygen conditions [48]. Here, an anaerobic DMSO-reductase coded by *dmsABC* was found in three of our *C*. *jejuni* water isolates (ST1275CC, ST683 and ST793). This DMSO-reductase has been shown to be common in *C*. *jejuni* isolates from humans and chicken [49] but uncommon in bovine isolates [49].

The majority of our isolates were able to form biofilm under the conditions tested (Fig 1). By forming biofilm, bacteria can create a protective environment for survival under harsh conditions [18]. As motility has been shown to be important for the formation of biofilm [19–21], the results from the biofilm assay were compared to the motility results. However, we could not see any correlations between the results. Although variations were seen in the motility genes between the *C*. *jejuni* water isolates, none of them could fully explain the differences in motility and biofilm formation. *PseE* mutations have earlier been shown to result in a non-motile phenotype [50], but here we could not see any motility loss for the isolates that seemed to lack an intact *pseE* gene. However, an intact *pseE* was only identified in the biofilm positive isolates suggesting that *pseE*-dependent flagellar modification is important for biofilm formation. In addition to motility, quorum sensing has been shown to affect the biofilm formation and studies have shown a decreased biofilm formation and motility in mutants lacking *luxS* [39,51]. Here, *luxS* was detected in four out of five water isolates that formed biofilm, but was lacking in the biofilm negative isolates. The swarming zones of the isolates were also compared with the genome sizes and the results showed that the isolate with the smallest genome had the highest motility and the isolate with the largest genome had the smallest swarming zone (Fig 2). These results may suggest that the *C*. *jejuni* that are less motile either contain more genetic material to compensate for the disadvantage or that the larger (heavier) genome directly and negatively impact the ability of the bacteria to move. Together, these findings suggest that the motility and biofilm phenotypes are multigenic and attributed to variations in a number of different genes.

The potential virulence of the *C*. *jejuni* water isolates was assessed in an *in vitro* infection model. The ability of the isolates to adhere to/invade and induce an immune response in a human colon cancer cell line was analyzed. All of the water isolates were able to both adhere/invade the cultured cells and to induce an IL-8 response, which suggests a potential to infect and cause inflammation in humans. However, as compared to the *C*. *jejuni* NCTC 11168 reference strain, the levels of adherence/invasion and IL-8 response were considerably lower. CDT is a toxin that has been shown to induce an IL-8 response [52] and in four of the *C*. *jejuni* water isolates, belonging to ST48 and ST683, the intact gene locus *cdtABC* was identified. The level of adherence/invasion for the isolates with intact *cdtABC* (ST48) varied, however, all the ST48 isolates induced the same level of IL-8 response (Fig 3). In one study the *in vitro* IL-8 response was not affected by CDT when the *C*. *jejuni* were able to adhere/invade [52], which might explain why isolates lacking CDT were able to induce similar levels of IL-8 as ST48 isolates. Together, these results suggest that the IL-8 response can be induced by different bacterial virulence mechanisms. As all isolates had intact genes for the putative virulence genes *ciaB*, *pldA*, *cadF* and *ceuE*, a more thorough analysis of expression levels of these genes might give an indication of the specific virulence strategy for each isolate.

A gene cluster coding for T6SS, a structure through which bacteria can deliver effector proteins into adjacent prokaryotic and eukaryotic cells [53], was identified in three of the *C*. *jejuni* water isolates (Table 2). The T6SS may be advantageous for the bacteria in several ways, e.g. to outcompete or to interact with other bacteria in the gut or in the environment [53]. However, as the T6SS gene cluster was not present in all of the water isolates, this structure does not seem to be critical for the survival in water.

The ST48 water isolates were assigned to LOS locus class B2 in accordance with a previous report [54]. *C*. *jejuni* with potential to sialylate LOS (LOS locus A, B and C) have been proposed to cause more severe symptoms [55] and also to adhere and invade cultured intestinal epithelial cell lines at a higher level than isolates without this characteristic [56,57]. In this study, no differences in the *in vitro* infections were seen between the isolates with or without the potential to sialylate their LOS. This is in line with our previous study [25] where *C*. *jejuni* with potential to sialylate LOS did not induce a higher IL-8 response than those without the ability to sialylate their LOS.

In conclusion, this is, to the best of our knowledge, the first study where *C*. *jejuni* isolated from water have been characterized using both whole genome sequencing and phenotypical assays. Although our collection of water isolates was limited, we found both characteristics common for all water isolates but also interesting genotypical and phenotypical differences between the isolates that might influence their potential to survive in the environment and transmit to human hosts.

## Supporting information

S1 Table RAST annotations of orthologues not shared by all *C*. *jejuni* water isolates. (DOCX)

S2 Table Annotations of plasmid genes found in *C*. *jejuni* ST48 water isolates from *C*. *jejuni* strain F38011. (DOCX)
