# Supplementary material for: Genomic and phenotypic characteristics of Swedish C. jejuni water isolates
Source: PLoS One. 2017 Dec 7;12(12):e0189222. doi: 10.1371/journal.pone.0189222 (PMC5720728; doi:10.1371/journal.pone.0189222)
Supplement: S1 Table — (DOCX) [file pone.0189222.s001.docx]

Table S1 RAST annotations of orthologues not shared by all C. jejuni water isolates

| **RAST annotation** | **Number of features** | **Comments** |
| --- | --- | --- |
| **Shared by ST48CC and ST1275CC** |  |  |
| - Aldehyde dehydrogenase A/Glycolaldehyde dehydrogenase | 1 | L-rhamnose utilization/methylglyxol metabolism |
| - Altronate dehydratase | 2 |  |
| - Fucose permease | 1 | L-fucose utilization |
| - Ferric siderophore system, periplasmic binding protein TonB | 1 | Also shared with VA48 (ST793) and VA49 (ST8853) |
| - Hemerythrin-like iron-binding protein | 1 | *Campylobacter* iron metabolism |
| - Hypothetical protein | 3 |  |
| - Membrane protein | 1 |  |
| - MFS Superfamily tartrate transporter | 1 |  |
| - Predicted metal-dependent hydrolase of the TIM-barrel fold | 1 | Blast in NCBI database yielded hit as amidohydrolase |
| - Putative lyase | 1 |  |
| - Small hydrophobic protein | 1 |  |
| - Transcriptional regulator, IclR family | 1 | Homogentisate pathway of aromatic compound degradation |
| - Lactam utilization protein LamB | 1 | Found in all *C. jejuni* water isolates. Frameshift in VA12 (ST1275CC), VA48 (ST793) and VA49 (ST8853). |
| **Shared by ST48CC and u.a.** |  |  |
| - Arsenic resistance gene cluster | 4 | Also in VA33 (ST683)*.* Premature stopcodon in *Acr3* coding for efflux pump protein disrupted and *arsP* fragmented (also in VA33) |
| - CdtABC | 3 | Also in VA33 (ST683) |
| - Putative amino acid activating enzyme | 1 | Shared with VA33 (ST683), VA48 (ST793) and VA49 (ST8853) |
| - 5,10-methylenetetrahydrofolate reductase | 1 | Shared with VA33 (ST683) |
| - Hypothetical protein | 1 | Shared with VA33 (ST683), VA48 (ST796) and VA49 (ST8853). Not in VA25. |
| - Possible sugar transferase | 1 | Blast in NCBI database yielded hit as *Campylobacter* invasion protein, CipA (Cj0685)c. Shared with VA33 (ST683), VA48 (ST796) and VA49 (ST8853). |
| **Shared by ST1275CC and u.a.** |  |  |
| - DmsoABC | 3 | Anaerobic respiratory reductases. Shared with VA33 (ST683) and VA48 (ST793) Disrupted in VA49 (ST8853). |
| - Highly acidic protein | 1 | Shared with VA33 (ST683), VA48 (ST793) and VA49 (ST8853). |
| - Hypothetical protein | 2 | Shared with VA33 (ST683), VA48 (ST793) and VA49 (ST8853). |
| - Lipopolysaccharide core biosynthesis protein LpsA | 1 | Shared with VA33 (ST683), VA48 (ST793) and VA49 (ST8853). Frameshift in VA12 and VA49. |
| - VgrG protein | 13 | Part of T6SS. Intact T6SS shared with VA48 (ST793) and VA49 (ST8853). |
| - IncF plasmid conjugative transfer protein TraG | 1 | Shared with VA33 (ST683), VA48 (ST793) and VA49 (ST8853). |
| **Shared only by u.a.** |  |  |
| - Hypothetical protein | 1 |  |
|  |  |  |
