# Supplementary material for: Genomic and phenotypic characteristics of Swedish C. jejuni water isolates
Source: PLoS One. 2017 Dec 7;12(12):e0189222. doi: 10.1371/journal.pone.0189222 (PMC5720728; doi:10.1371/journal.pone.0189222)
Supplement: S2 Table — (DOCX) [file pone.0189222.s002.docx]

Table S2 Annotations of plasmid genes found in *C. jejuni* ST48 water isolates from *C. jejuni* strain F38011.

| **Annotation** | **Size (bp)** |
| --- | --- |
| Hypothetical protein | 1320 |
| Hypothetical protein | 306 |
| 3-deoxy-manno-octulosonate cytidylyltransferase | 720 |
| Threonine synthase | 1413 |
| Tetraacyldisaccharide 4’-kinase | 927 |
| NAD synthase | 741 |
| Betalactamase | 597 |
| 7-alpha-hydroxysteroid dehydrogenase | 780 |
| Dihydrodipicolinate synthase | 897 |
| Protease | 1251 |
| Diguanylate cyclase | 1059 |
| ABC transporter permease | 1743 |
| Cysteinyl-tRNA synthetase | 1389 |
| Membrane protein | 1452 |
| ATPase | 1860 |
| ruvA | 552 |
| D-alanine--D-alanine ligase | 1041 |
| Prevent-host-death protein | 216 |
| 2-hydroxy-6-oxohepta--2,4-dienoate hydrolase | 720 |
| UDP-N-acetylmuramoyl-tripeptide--D-alanyl-D-alanine ligase | 1428 |
